# Surface Evolution of Nano-Textured 4H–SiC Homoepitaxial Layers after High Temperature Treatments: Morphology Characterization and Graphene Growth

**DOI:** 10.3390/nano5031532

**Published:** 2015-09-18

**Authors:** Xingfang Liu, Yu Chen, Changzheng Sun, Min Guan, Yang Zhang, Feng Zhang, Guosheng Sun, Yiping Zeng

**Affiliations:** 1 Key Laboratory of Semiconductor Materials Science, Institute of Semiconductors, Chinese Academy of Sciences, P.O. Box 912, Beijing 100083, China; E-Mails: guanmin@semi.ac.cn (M.G.); zhang_yang@semi.ac.cn (Y.Z.); fzhang@semi.ac.cn (F.Z.); gshsun@semi.ac.cn (G.S.); ypzeng@semi.ac.cn (Y.Z.); 2 Semiconductor Lighting Technology Research and Development Center, Institute of Semiconductors, Chinese Academy of Sciences, P.O. Box 912, Beijing 100083, China; 3 Tsinghua National Laboratory for Information Science and Technology, Tsinghua University, Beijing 100084, China; E-Mail: czsun@tsinghua.edu.cn

**Keywords:** nano-textured, 4H–SiC, morphology, graphene, evolution

## Abstract

Nano-textured 4H–SiC homoepitaxial layers (NSiCLs) were grown on 4H–SiC(0001) substrates using a low pressure chemical vapor deposition technique (LPCVD), and subsequently were subjected to high temperature treatments (HTTs) for investigation of their surface morphology evolution and graphene growth. It was found that continuously distributed nano-scale patterns formed on NSiCLs which were about submicrons in-plane and about 100 nanometers out-of-plane in size. After HTTs under vacuum, pattern sizes reduced, and the sizes of the remains were inversely proportional to the treatment time. Referring to Raman spectra, the establishment of multi-layer graphene (MLG) on NSiCL surfaces was observed. MLG with *sp*^2^ disorders was obtained from NSiCLs after a high temperature treatment under vacuum at 1700 K for two hours, while MLG without *sp*^2^ disorders was obtained under Ar atmosphere at 1900 K.

## 1. Introduction

Silicon carbide (SiC)-derived carbon, especially graphene, which can be grown on SiC by thermal decomposition at high temperature under vacuum [1], has attracted intense interest in recent years. Graphene is a kind of planar honeycomb structure, formed by carbon atoms by means of *sp*^2^ hybrid chemical bonds, whose unique electronic properties have been predicted in theory for decades [2]. Since graphene has been prepared experimentally [3,4,5,6,7,8,9], many of its properties have been verified [10,11,12,13].

Graphene preparation via high temperature treatments (HTTs) possesses at least two merits. First, thermal decomposition is a relatively simple process, since it can be performed at a wide temperature range, e.g., 1400~2000 K under high or medium vacuum, even at Ar ambient conditions [7]. Second, after graphene growth, the SiC host can be used as the substrate for graphene device fabrication, thus avoiding film assembly and/or transfer as necessitated by graphene production methods without substrate, via solution [14] or mechanical exfoliation [3,15]. In early stages, semi-insulating hexagonal SiC substrates without miss-cut surfaces, *i.e.*, on-axis surfaces, were chosen for graphene growth; this choice was determined by the fact that commercially available, semi-insulating substrates may thus be used for electronic isolation, while continuous graphene film growth is anticipated with on-axis substrates but not with miss-cut ones, since there are steps on the surfaces of the miss-cut substrates [16] which perhaps make graphene film discontinuous. In order to explore new perspectives for graphene sciences, n-type, hexagonal SiC films with miss-cut surfaces [17,18] and cubic SiC films, grown heteroepitaxially on silicon substrates [19,20], were also investigated.

In addition to growth on flat, rigid substrates to facilitate the planar device fabrication process, methods have been found to combine graphene with nano-materials, forming nanostructures which are expected for use in potential applications in nanoelectronics and optoelectronics [21]. Although much research on graphene growth has been performed on flat SiC surfaces [22,23], structured SiC surfaces [24], and discrete SiC particles [25], there is a dearth of research attending to surface evolution of nano-textured patterns on rigid SiC after high temperature treatments. For the first time, we reveal surface evolution and graphene growth aspects of nano-textured homoepitaxial 4H–SiC layers (NSiCLs) after high temperature treatments. These results will be useful for future nano, SiC-derived graphene growth.

## 2. Results and Discussion

**Figure 1:**
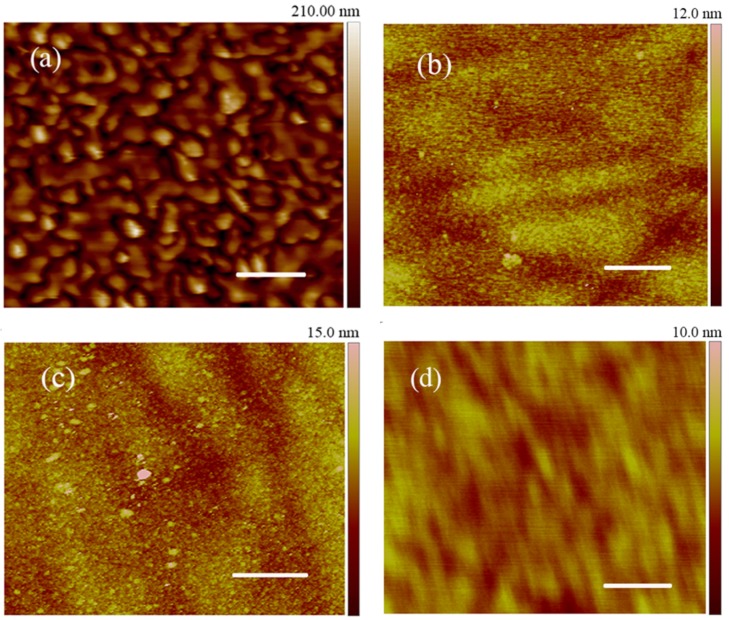
AFM morphologies of (**a**) NsiCL (Nano-textured 4H–SiC homoepitaxial layers), graphene (**b**) G-1h-1700K, (**c**) G-2h-1700K and (**d**) G-1h-1900K. Scale bars: 2 μm.

Due to high density nano-terraces on the epitaxial surfaces, which guide epitaxial growth in the so-called “step-control growth mechanism”, 4H–SiC homoepitaxial layers with smooth and uniform morphologies are usually grown on 4H(0001) substrates by low pressure chemical vapor deposition (LPCVD) [26,27]. According to the said mechanism, homoepitaxial growth occurs in two-dimensional (2D) mode, while the homoepitaxial layers grow on the substrate layer by layer, often yielding a moderate growth rate of 1–2 μm/h. However, when precursor flow increases, the growth rate rises to 40–120 μm/h for fast growth, disrupting the step-control growth mechanism, and often yielding epitaxial layers of rough morphology with microstructures [28]. At fast growth mode, there are plenty of SiC species simultaneously reaching their growth sites, usually the kinks in the terraces, which leads to disordered terrace growth. When terraces are discrete and tilted with each other at the start of epitaxial growth, nano-textures form whose feature sizes increase with the increased thickness of the epitaxial layer. Figure 1a shows the atomic force microscopy (AFM) morphology of a nano-textured, 4H–SiC homepitaxial layer. Although the surface of the epitaxial layer appears mirror-like under Nomarski optical microscopy, it is obvious to see that there are nano-features on the surface. AFM morphologies of NSiCLs differ from those of homoepitaxial layers grown by conventional configuration with a moderate growth rate; the latter often show no features, or show wide terraces which are almost parallel with each other [29]. The AFM morphologies of NSiCLs also differ from those of thick homoepitaxial layers grown in fast mode; the surfaces of thick layers are often rough, with terraces bunched and disordered [28].

Nano-textures evolve after high temperature treatments. Figure 1b,c displays AFM morphologies of samples G-1h-1700K and G-2h-1700K, treated at 1700 K under medium vacuum of 10^−3^ Pa for one hour and two hours, respectively (please find sample specifics in the experimental section). Nano-features still appear on the surfaces of the mentioned samples; however, sizes of the features are miniscule compared to those of NSiCLs. Although binary compound SiC is chemically inert and it is difficult to break the Si–C bonds, when subjected to high temperature treatment, silicon and carbon bonds break from surface level to a few monolayers beneath, and silicon atoms detach from the SiC surface while the remaining carbon atoms rebond with each other and rearrange on the surface. Nano-texture surfaces will be more active for decomposition than flat surfaces due to their larger surface area. They are also especially reactive on the edges since there are more unsaturated bonds and fewer bonds that have to break to detach an atom from an edge. At high temperature, those Si–C bonds at lower-energy barriers will break more easily, while unbound SiC molecules or clusters will detach from surfaces until all lower-energy barrier bonds are broken. In the end, nano-texture surfaces will lose many SiC fractures, becoming smoother with smaller features, as shown in Figure 1b–d. Figure 1d depicts the AFM morphology of sample G-1h-1900K, treated at 1900 K under Ar atmosphere, whose morphology is stripe-like, different from those of G-1h-1700K and G-2h-1700K, which are grainy and similar to NSiCLs.

**Figure 2:**
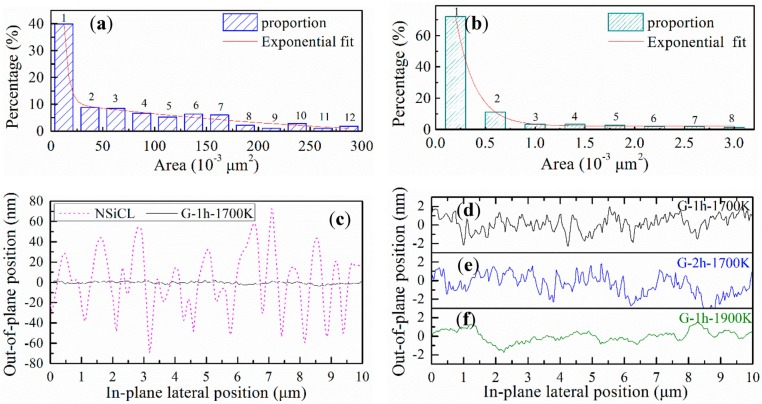
Statistical histograms of (**a**) NSiCL and (**b**) graphene G-2h-1700K, and line-sectional analyses of (**c**) NSiCL and graphene G-1h-1700K, (**d**) G-1h-1700K, (**e**) G-2h-1700K and (**f**) G-1h-1900K.

Feature sizes of those samples and their evolution are illustrated in Figure 2. NSiCLs develop at about micron scale in-plane and at nano scale out-of-plane, all of which can be roughly divided into 12 categories, labeled 1–12 in sizes ranging from 10 to 290 (the unit is 10^−3^ square microns, as it is below), as shown in Figure 2a. The most miniscule category (labeled 1) comprises the largest sample population at about 40%. Categories 2–7, with sizes 30~160, are evenly distributed, containing a collective 50% of total samples, while the remaining 10% are evenly distributed among the remaining categories. To describe this distribution, the feature sizes of NSiCL can be fitted to a three-stage, exponential decay curve, *f*(*x*), expressed as:

(1) $$f\left(x\right)=a_{0}+\overset{3}{\underset{i=1}{\sum }}a_{i}e^{-\left(x-x_{0}\right)/t_{i}}$$

where *a*_0_, *a_i_*, *t_i_*, and *x*_0_ are fitting constants, and the variable parameter *x* is the in-plane lateral position.

Apart from feature sizes, the morphologies of graphene G-1h-1700K and G-2h-1700K are similar to each other and to that of NSiCL (Figure 1). We chose graphene G-2h-1700K (the corresponding AFM image is Figure 1c) as the sample for feature size evaluation. The feature sizes of graphene occur reduced by nearly two orders of magnitude (Figure 2b) when compared to their corresponding raw NSiCLs (Figure 2a), while the appropriate number of categories is also reduced to eight. A three-stage, exponential decay curve similar to the formula in Equation (1) can be used to fit the population data. The first stage (labeled 1) is the category with plane feature sizes of about 0.2–0.3, which correspond to more than 70% of samples, while the second stage comprises only one category (labeled 2) holding about 10% of samples. The final stage, containing categories labeled 3–8, is also evenly-distributed, and possesses less than 20% of samples.

To further investigate the in-plane and out-of-plane feature sizes of NSiCL and graphene, we performed line-sectional analyses on AFM images. Using a horizontal line across the middle of the images, sectional data were extracted from AFM images in Figure 1, where the undulating topographies of NSiCL and graphene with position changes are clearly shown in Figure 2c–f. The in-plane feature sizes are consistent with results from the statistical distribution analyses (Figure 2a,b), while the out-of-plane feature sizes of NSiCL and graphene are significantly different. The former are in a range of 20 nm–140 nm, while the latter only several nanometers. The amplitude of the out-of-plane graphene features is so small that they mostly form a straight line on the sectional analysis graph when compared to that of the NSiCL (Figure 2c). The feature size profiles of graphene G-1h-1700K and G-2h-1700K are almost the same, except that the amplitude of the out-of-plane feature sizes of G-1h-1700K (Figure 2d) is slightly smaller than those of G-2h-1700K (Figure 2e). Compared to the rough profile of G-1h-1700K (or G-2h-1700K), which is composed of many zig-zag curves forming an undulating outline, the profile of G-1h-1900K is smoother, with each outline composed of none to a few zig-zag curves (Figure 2f).

**Figure 3:**
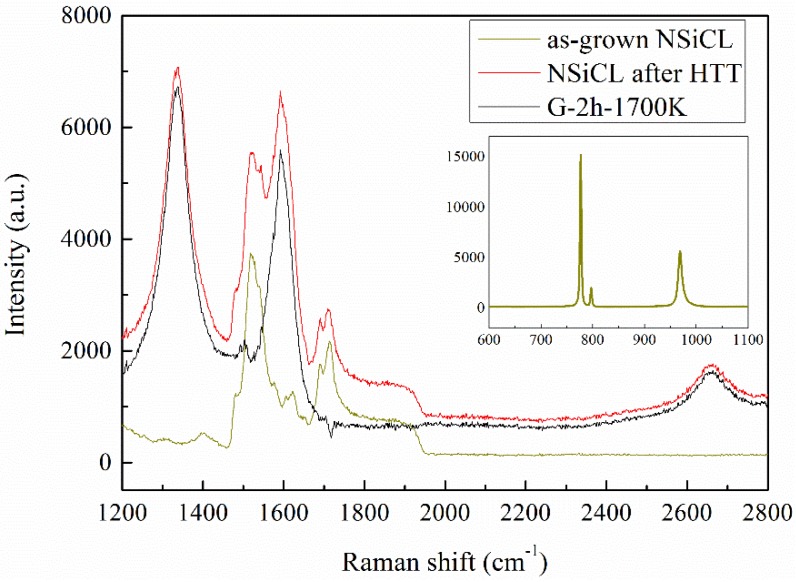
Raman spectra of the as-grown NSiCL and the NSiCL after a high temperature treatment (HTT), respectively, and the derived graphene of sample G-2h-1700K. Inset: Raman peaks of TO mode and LO mode of the as-grown NSiCL.

We used Raman spectra to judge whether NSiCL is a 4H polytype, as well as find layer and defect information of the obtained graphene. Figure 3 shows respective Raman spectra of as-grown NSiCL and NSiCL subjected to high temperature treatment, respectively, and the derived graphene (sample G-2h-1700K). Judging from all the Raman spectra, the as-grown NSiCLs are the 4H polytype, as indicated by Raman peaks of the transverse optic (TO) mode and longitudinal optic (LO) mode, wholly belonging to the 4H polytype [30], while no other peak appears, such as the 3C polytype. After high temperature treatment, the Raman spectrum of NSiCL has three additional peaks in the range of 1200 cm^−1^–2800 cm^−1^. Usually named D mode, G mode, and 2D mode, respectively, peaks located at 1350 cm^−1^, 1580 cm^−1^, and 2700 cm^−1^ relate to graphene signals [31], which indicate that graphene has been obtained from NSiCL after high temperature treatment. To clearly illustrate the Raman spectrum of graphene, the SiC Raman signals are often subtracted [32].

**Figure 4:**
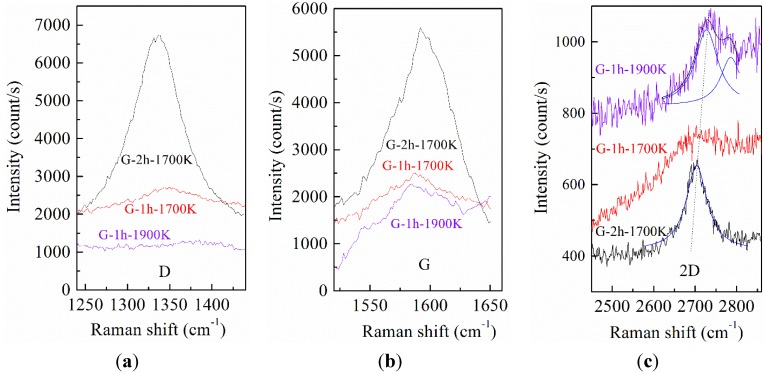
Raman spectra of graphene G-1h-1700K, G-2h-1700K, and G-1h-1900K: (**a**) D peaks, (**b**) G peaks and (**c**) 2D peaks.

**Figure 5:**
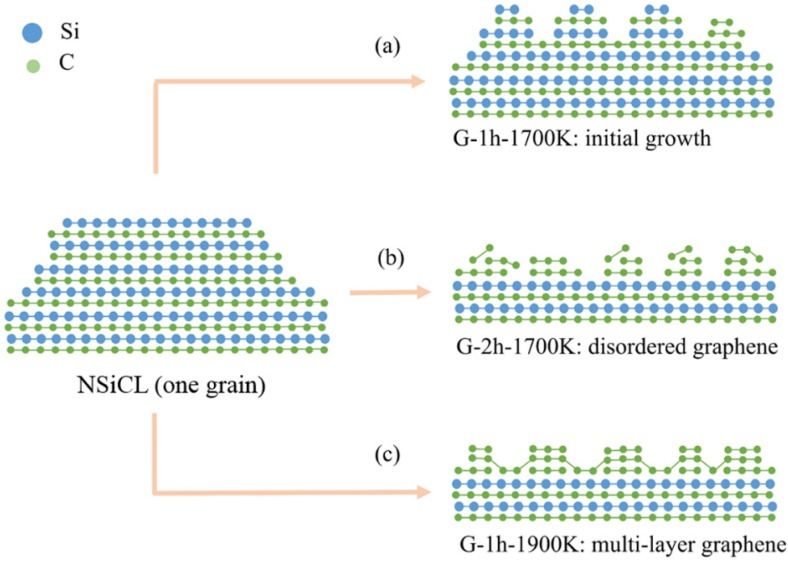
Schematic illustration for surface evolution of NSiCL (one grain extracted from the nano-textured SiC layer as an example) and corresponding graphene growth. After subjection to high temperature treatments (HTTs), nano-patterns on the SiC layer were reduced. Here, (**a**) G-1h-1700K; (**b**) G-2h-1700K; and (**c**) G-1h-1900K are, respectively, instances of initial growth, disordered graphene growth, and multi-layer graphene growth according to various treatment techniques.

Figure 4 shows D modes, G modes, and 2D modes of Raman spectra of graphene G-1h-1700K, G-2h-1700K, and G-1h-1900K, respectively, where NSiCL Raman signals were subtracted before graphene Raman spectra composition [32]. All named Raman peaks of G-1h-1700K appear but are barely distinguishable; we are therefore not sure if, or to what extent, graphene was grown on NSiCL in this case. It is more practical to speculate that graphene growth initiates at this growth condition (Figure 5a), and that graphene will grow on NSiCL with increased growth time. In the case of G-2h-1700K, we increased only growth time, while keeping other growth conditions the same as those of G-1h-1700K, and graphene was indeed grown on NSiCL (Figure 5b).

The 2D mode is the fingerprint signal by which graphene can be identified [31]. The frequency intensity ratio of the 2D-to-G peak (denoted as *I*_2D_/*I*_G_) is used to identify the number of graphene layers [33]. Raman spectra with *I*_2D_/*I*_G_ ~ 2 indicate monolayer graphene, while those with 1 < *I*_2D_/*I*_G_ < 2 indicate bilayer graphene [6]. The values of *I*_2D_/*I*_G_ for our samples are all less than 1, indicating that the obtained graphene are multi-layer graphene (MLG) (Figure 5c).

The full width at half maximum (FWHM) of the 2D peak is another indicator of graphene quality [34]. G-2h-1700K shows a symmetrical 2D peak, which appears at 2702 cm^−1^, plus the 2D peak can be fitted by a one-peak Lorentz fitting curve (Figure 4c). For a single layer of graphene, the cut-off FWHM of the 2D peak is 30 cm^−1^ [35]. The 2D FWHM increases with an increased number from mono-layer to multi-layer. For bilayer graphene, the 2D FWHM is found to be 43–53 cm^−1^. For trilayer graphene, the 2D FWHM is found to be 56–63 cm^−1^ [36]. The 2D FWHM of G-2h-1700K is 61 cm^−1^, obtained from the Lorentz fitting curve. Judging from the above analyses, G-2h-1700K is trilayer graphene. However, the Raman peaks of G-1h-1700K and G-1h-1900K are not readily suitable for Lorentz fitting. G-1h-1700K is an instance of initial graphene growth; the Raman peaks of it are barely distinguishable, so the fitted values are not reliable. G-1h-1900K displays a more complex peak than G-2h-1700K, and the 2D peak of G-1h-1900K is asymmetrical with an attendant peak located at 2784 cm^−1^. We speculate the possible origin of this shape is correlated with the topography of the substrate surface. For example, the Raman spectrum is collected over several nano-textured regions of the graphene surface, subjected to different amounts and types of strain, as those of Robinson *et al.* were reported [37]. The D peak is associated with *sp*^2^ defects or local disorders [38]. Graphene G-2h-1700K is therefore more defective than graphene G-1h-1900K in that no D peak appears in G-1h-1900K Raman spectrum.

Figure 5 is a schematic representation of the surface evolution and graphene growth on a grain of the nano-textured SiC film during high temperature annealing. Here, the routes of (a), (b) and (c) in Figure 5, which stands for instances of initial growth (G-1h-1700K), disordered graphene growth (G-2h-1700K), and multi-layer graphene growth (G-1h-1900K), respectively, according to various treatment techniques. This schematic includes interpretations for each of our samples.

When graphene is grown on the Si-face of SiC(0001), it often forms an interfacial buffer layer of carbon between the Si face and graphene [39]. This is believed to be at the origin of the Bernal stacking in multi-layer graphene on the Si face. Such a buffer layer is not present for other SiC orientations, *i.e.*, the C-face [40] and the non-polar faces [41]. Furthermore, it has been recently shown that the buffer layer is locally delaminated from oblique facets (11$\bar{2}$n) of off-angle Si-face SiC wafers [42]. In this study, graphene has been grown on the Si-face of SiC(0001), hence it would form an interfacial buffer layer; however, the SiC surface is nano-textured, with oblique facets (11$\bar{2}$n), and, therefore, the buffer layer is locally delaminated [42]. This result is different than in previous studies [4,7,43], in which flat 4H–SiC substrates were used.

## 3. Experimental Section

Nano-textured 4H–SiC homoepitaxial layers (NSiCLs) with nominal thickness and doping concentration of 620 nm and 5.8 × 10^15^ cm^−3^, respectively, were grown on commercially available, on-axis 4H–SiC(0001) substrates, using low pressure chemical vapor deposition. In order to obtain surfaces with nano-textured morphology, an unconventional growth technique was adopted. Before epitaxial growth, 4H–SiC(0001) substrates were etched under H_2_ atmosphere at 1600 K for one hour to prepare clean and smooth surfaces. After etching, the temperature was raised to 1850 K rapidly, while SiHCl_3_ and C_2_H_4_ as precursors, diluted in highly pure H_2_ (99.999%), were fed into the chemical vapor deposition (CVD) chamber for epitaxial growth. Precursors were turned off after a 30 s growth period, and the 4H–SiC samples cooled down to room temperature naturally under H_2_ atmosphere. Pressures were kept at 10^4^ Pa during the whole process.

NSiCLs were subjected to high temperature treatments in an induction furnace. Samples were treated at 1700 K under medium vacuum of 10^−3^ Pa for one and two hours, respectively, while some samples were treated at 1900 K under Ar atmosphere of 1.013 × 10^5^ Pa (1 atm) for one hour. The corresponding samples are denoted as G-1h-1700K, G-2h-1700K, and G-1h-1900K, respectively.

Morphologies of both NSiCL and graphene were characterized by atomic force microscopy (AFM, Veeco Instruments, NewYork, NY, USA), while morphology features were characterized by statistical histogram and sectional analyses. Raman scattering spectra (Horiba HR800, Horiba Jobin Yvon, Paris, France) were used to evaluate graphene quality, and were performed at room temperature using an He-Ne laser operating at the 632.8 nm line mode with a confocal microscope at about 1 μm spatial resolution.

## 4. Conclusions

In summary, nano-textured 4H–SiC homoepitaxial layers (NSiCLs) were grown on 4H–SiC(0001) by an unconventional epitaxial method using low pressure chemical vapor deposition, producing as-grown NSiCLs with feature sizes of about 0.01–0.29 square microns. After subjection to different high temperature treatments, feature sizes of the NSiCLs were reduced, while remaining features were at least two orders of magnitude smaller than on as-grown NSiCLs. Graphene was grown on these 4H–SiC homoepitaxial layers. The initial growth of graphene took place after a high temperature treatment at 1700 K under medium vacuum of 10^−3^ Pa for one hour, while multi-layer graphene (MLG) grew in two hours. MLG without *sp*^2^ disorders was also grown under argon once NSiCL was treated at the higher temperature of 1900 K. This investigation produces an avenue of refined technique for graphene production, expanding the pathway for future nano, SiC-derived graphene growth.
